# The rise of ocean giants: maximum body size in Cenozoic marine mammals as an indicator for productivity in the Pacific and Atlantic Oceans

**DOI:** 10.1098/rsbl.2016.0186

**Published:** 2016-07

**Authors:** Nicholas D. Pyenson, Geerat J. Vermeij

**Affiliations:** 1 Department of Paleobiology, National Museum of Natural History, Smithsonian Institution, PO Box 37012, Washington, DC 20013-7013, USA; 2 Department of Paleontology, Burke Museum of Natural History and Culture, Seattle, WA 98195, USA; 3 Department of Earth and Planetary Sciences, University of California, Davis, One Shields Avenue, Davis, CA 95616, USA

**Keywords:** gigantism, marine mammals, predators, escalation, fossil record

## Abstract

Large consumers have ecological influence disproportionate to their abundance, although this influence in food webs depends directly on productivity. Evolutionary patterns at geologic timescales inform expectations about the relationship between consumers and productivity, but it is very difficult to track productivity through time with direct, quantitative measures. Based on previous work that used the maximum body size of Cenozoic marine invertebrate assemblages as a proxy for benthic productivity, we investigated how the maximum body size of Cenozoic marine mammals, in two feeding guilds, evolved over comparable temporal and geographical scales. First, maximal size in marine herbivores remains mostly stable and occupied by two different groups (desmostylians and sirenians) over separate timeframes in the North Pacific Ocean, while sirenians exclusively dominated this ecological mode in the North Atlantic. Second, mysticete whales, which are the largest Cenozoic consumers in the filter-feeding guild, remained in the same size range until a Mio-Pliocene onset of cetacean gigantism. Both vertebrate guilds achieved very large size only recently, suggesting that different trophic mechanisms promoting gigantism in the oceans have operated in the Cenozoic than in previous eras.

## 1. Introduction

Tracking primary productivity in the oceans over time is one of the major goals of palaeoceanography and palaeoecology because the rate of carbon fixation by marine primary producers depends on global patterns of ocean circulation, climate and ecosystem structure that have changed markedly over geologic time [1,2]. Despite the lack of direct metrics for primary productivity in past oceans, one promising indirect avenue is to measure the maximum body sizes of consumers (herbivores and planktivores) that benthic seaweeds and benthic photosymbiotic animals can sustain [3,4]. In this regard, consumer body size is a direct consequence of the available primary productivity within the sphere of influence of an individual consumer [3]. We argue that, over geologic time, patterns in the maximum body size of consumers indicate temporal changes in the enabling factor of primary productivity on the seafloor and in the pelagic zone.

Here, we evaluated the maximum sizes of two ecological groups of large, metabolically active marine consumers (bottom-feeding herbivorous and pelagic filter-feeding mammals) in the North Pacific and Atlantic oceans, two basins whose marine vertebrate record is adequate and well documented [5]. Vermeij [3] indicated that the history of gigantism in bottom-feeding molluscs, among several ecological guilds and trophic groups, differed between these two basins, and that maximum size became greater in the temperate North Pacific during or shortly before the Pliocene (5.3–2.6 Ma). Because marine vertebrates occupy broad geographical ranges, inferences of past primary productivity based on their maximum body sizes probably reflect global rather than more regional patterns chronicled by molluscs. Furthermore, temporal patterns in filter-feeding vertebrates should reflect productivity in the open ocean, whereas those of herbivores should reflect coastal productivity. Our findings imply a sharp rise in oceanic productivity and coastal North Pacific productivity during the Mio-Pliocene boundary, raising questions about the possible mechanisms underlying this surprisingly late increase.

## 2. Material and methods

We compiled maximal body size for two guilds of marine mammals in both the North Pacific and North Atlantic Ocean basins during the Cenozoic. We restricted our search to fossil-bearing rock units of the western and eastern coasts of these basins, while excluding fossils from the Mediterranean region and elsewhere (see electronic supplementary materials). We identified the largest single individual specimen known, either published or in a museum collection, binned by sub-epoch. Although these bins are unequal lengths of geologic time, this coarse scale permitted inter-basinal and cross-taxonomic comparisons. Known biasing factors in the fossil record (e.g. rock area, collecting efforts) do not appear to distort the diversity records of these groups, nor do they display strong ‘pull of the Recent’ signals (see electronic supplementary material, figure S1).

First, we examined large marine herbivorous mammals, comprising Sirenia and Desmostylia [6,7]. Comparability among fossil taxa in this guild is challenging because intact, associated skeletons are relatively rare and body plans differ sufficiently to prevent using traditional comparative proxies (i.e. post-Eocene sirenians lack weight-bearing hind limbs). We used cranial length as a proxy for body size, based on the strong allometric correlations known for sirenians [6]; we presume that similar allometries constrain desmostylian feeding ecology [7], given the ecomorphologic similarities in the rostrum and dentition of desmostylians and aquatic sloths (*Thalassocnus* spp.), and to a lesser extent, sirenians [8]. Although desmostylians probably retained some degree of terrestrial locomotion, multiple lines of evidence place their feeding palaeoecology firmly in aquatic environments [7], and thus in direct competition with sirenians, until the Late Neogene. Second, we collected skull width on mysticete cetaceans, which is a reliable size proxy [9] for the largest and exclusive members of the mammalian filter-feeding guild. We did not include so-called ‘toothed’ stem mysticetes in our dataset because it remains unclear whether they belonged to the same filter-feeding guild as baleen-bearing mysticetes (Eomysticetidae and crown-ward mysticetes).

## 3. Results and discussion

**Figure 1.**
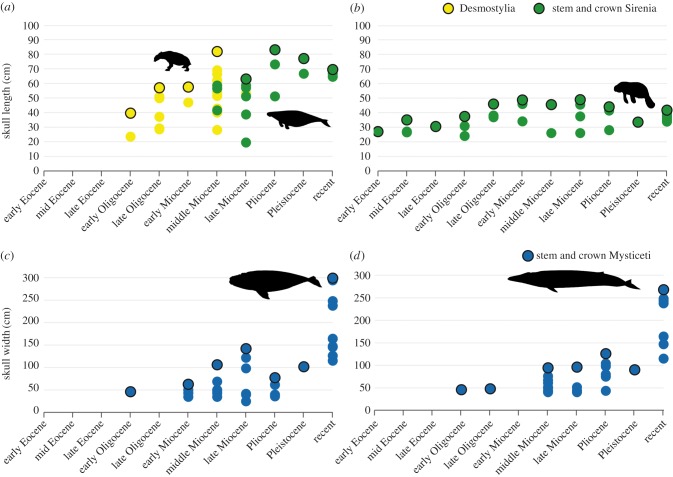
(*a*,*b*) Maximal body size in North Pacific and North Atlantic marine mammal herbivores, and (*c*,*d*) similarly for mammalian filter-feeders, during the Cenozoic. PhyloPics of herbivores, except *Hydrodamalis*, by Steven Traver.

While marine mammal herbivores quickly attained maximum size in both ocean basins, this guild never achieved the large body sizes of mysticetes (figure 1 and table 1). In the North Pacific, the late and slight rise in body size in the Plio-Pleistocene is entirely attributable to Steller's sea cow (*Hydrodamalis gigas*) and fossil hydrodamalines [10], which postdate the extinction of desmostylians. While this rise may reflect an adaptive response to colder conditions at high latitude, desmostylians did attain similar sizes at temperate latitudes in the Mid Miocene (figure 1). North Atlantic sirenians never attained the maxima thresholds in the North Pacific. There are strong geographical differences in the maxima of this guild because fossil sirenians in the North Atlantic are primarily subtropical and tropical and associated with seagrasses [11], especially in the western Atlantic and Caribbean region, whereas herbivores in the North Pacific span temperate to sub-polar latitudes. By contrast, mysticetes remained within a narrow body size range until the Plio-Pleistocene, with marked size increases driven by the lineage leading to blue whales (*Balaenoptera musculus*), which lack a fossil record, and right whales (*Eubalaena* spp.), including a smaller Late Miocene relative, *Eubalaena shinshuensis*, from Japan (see electronic supplementary material, table S1). While not all mysticetes feed at the same trophic level, we argue that the high variability in their diet [12], over geologic time and geography, minimizes these differences and essentially averages them across the entire guild.

**Table 1.** Maximal body size in North Pacific and North Atlantic marine mammal herbivores and mammalian filter-feeders during the Cenozoic. Measurements show cranial dimensions in centimetres; see the text and electronic supplementary material for details and data.

	North Pacific				North Atlantic			
	marine herbivore guild		filter-feeding guild		marine herbivore guild		filter-feeding guild	
recent	69.5	Sirenia	299	Mysticeti	41.7	Sirenia	268	Mysticeti
Pleistocene	77	Sirenia	101.6	Mysticeti	33.5	Sirenia	90.2	Mysticeti
Pliocene	83	Sirenia	85	Mysticeti	44	Sirenia	126	Mysticeti
late Miocene	63	Sirenia	160	Mysticeti	48.9	Sirenia	96.2	Mysticeti
mid Miocene	81.8	Desmostylia	106	Mysticeti	45.5	Sirenia	94.5	Mysticeti
early Miocene	57.5	Desmostylia	62.5	Mysticeti	48.7	Sirenia	0	none
late Oligocene	57	Desmostylia	0	none	45.9	Sirenia	48	Mysticeti
early Oligocene	39.6	Desmostylia	46	Mysticeti	37.4	Sirenia	46	Mysticeti
late Eocene	0	none	0	none	30.5	Sirenia	0	none
mid Eocene	0	none	0	none	35	Sirenia	0	none
early Eocene	0	none	0	none	27	Sirenia	0	none

Generally, our findings fit with previous work showing a dramatic rise in maximal body size of vertebrate filter-feeders and other marine mammals, especially after the Mio-Pliocene boundary [9,13,14]. Data on plankton-feeding molluscs do not indicate a sharp increase in maximum size from the Pliocene to the Pleistocene as the mysticete data do, but instead show an increase in the later Miocene and Pliocene, especially in the North Pacific, North Atlantic and tropics [3]. The maximum sizes of suspension-feeding molluscs reflect primary productivity of coastal plankton, whereas those for mammals integrate productivities on an oceanic scale, from near- to offshore. Based on modern stranding records [15], there is no reason to expect biases against pelagic fossil marine mammals or oversampling of near-shore ones (i.e. habitats of sirenians and desmostylians [7]). Inter-oceanic differences in maximum size, and therefore by inference in pelagic productivity, are less severe than differences among basins in coastal benthic size and productivity.

Our results are consistent with the hypothesis that benthic primary production was higher in the temperate North Pacific than in the subtropical Atlantic from at least Early Oligocene time, a pattern inferred for temperate molluscs [4,16]. The North Pacific is the basin of origin for kelps (Laminariales), which include the largest marine plants (*Nereocystis* and *Macrocystis*). Kelps, especially the two large-bodied genera, grow rapidly, transport nutrients through phloem-like medullary tissues and are adapted to intense herbivory [4]. The coincident rise of kelps and large marine herbivorous mammals indicates a positive escalation, culminating in the relatively recent origin of *Nereocystis* and *Macrocystis,* probably in response to intense herbivory by desmostylians and sirenians [10]. The seagrasses on which Atlantic sirenians feed are also productive, but the plants are smaller and rates of production are lower than that for kelps. No marine mammalian herbivores have evolved in the temperate North Atlantic or in most of the Southern Hemisphere, despite the spread of Laminariales to these basins and of *Macrocystis* to Australasia and western South America [17]. Mio-Pliocene-age marine sloths (*Thalassocnus* spp.) that evolved in Peru and Chile never attained the large body sizes of North Pacific sirenians and probably fed on seagrasses [8]. While our data do not focus on the Southern Hemisphere, the improving fossil record of South American fossil marine vertebrates (e.g. [18,19]) will soon provide sufficient basis for such inter-hemispheric comparisons.

The general increase in maximum body size to a broad Neogene peak in herbivorous mammals appears to coincide with a rise in near-shore primary benthic productivity, especially in the North Pacific [3,4], where exceptionally large and productive seaweeds arose and diversified. Primary production near-shore was stimulated by increased runoff from the tectonically highly active continents during the Neogene [16]. The all-time maximum size of filter-feeding mammals coincides with the onset of large-scale glaciation in the Pleistocene, which together with continuing intense erosion and chemical weathering invigorated ocean circulation and productivity worldwide [20]. Intriguingly, mammalian body size patterns at sea are far delayed from those on land, where maxima were achieved relatively quickly following the end of the Cretaceous, across different lineages and continents [13].

We argue that maximal body size increases among marine mammals were enabled by increasing marine productivity in benthic and pelagic ecosystems during the Neogene. The largest herbivorous and filter-feeding marine animals are mammals, whose metabolic rates are higher than those of functionally equivalent ectothermic fishes [21], which mysticetes replaced as dominant guild members at least by the Neogene [22]. Cenozoic body size patterns may not be comparable with those in Mesozoic oceans, where the largest suspension feeders were smaller than their Cenozoic counterparts [22], and where marine herbivores did not evolve until the Cretaceous [23]. Although the largest guild members studied here outstripped Mesozoic body size maxima for marine reptiles, widespread modes of hypercarnivory within Mesozoic feeding guilds point to major differences in community size structuring, probably underpinned by differences in trophic structuring [24]. Such patterns may be broader in the fossil record than previously recognized [25].

## Supplementary Material

ESM text

## Supplementary Material

ESM spreadsheet

## Data accessibility

All additional data are in the electronic supplementary material file.
