# Supplementary material for: The rise of ocean giants: maximum body size in Cenozoic marine mammals as an indicator for productivity in the Pacific and Atlantic Oceans
Source: Biol Lett. 2016 Jul;12(7):20160186. doi: 10.1098/rsbl.2016.0186 (PMC4971165; doi:10.1098/rsbl.2016.0186)
Supplement: ESM text [file rsbl20160186supp1.docx]

Electronic Supplemental Material

THE RISE OF GIANTS: Maximum body size in cenozoic marine MAMMALS as aN INDICATOR for marine productivity in the pacific and atlantic oceans

Nicholas D. Pyenson^1,2*^ and Geerat J. Verneij^3^

^1^Department of Paleobiology, National Museum of Natural History, Smithsonian Institution, P.O. Box 37012, Washington DC 20013-7013, USA

^2^Department of Paleontology, Burke Museum of Natural History and Culture, Seattle, Washington, 98195, U.S.A

^2^Department of Earth and Planetary Sciences, University of California, Davis, One Shields Avenue, Davis, CA  95616

*Correspondence: Nicholas D. Pyenson [pyensonn@si.edu](mailto:pyensonn@si.edu)

**Table of Contents for Supplemental Materials and Methods**

**1. Specimen-based approach p. 2**

**2. Data sources p. 3**

**3. Biases p. 6**

**4. Supplemental text references p. 9**

**5. Institutional abbreviations cited in the dataset p. 11**

**6. References cited in the dataset p. 12**

**7. Figure S1 p. 21**

**Supplemental Materials and Methods**

**1. Specimen-based approach**

We compiled specimen-based records for the maximal body sizes of two guilds of marine mammals in both the North Pacific and North Atlantic ocean basins during the Cenozoic. In this study, we explicitly tied maxima records for different fossil and living marine mammals to natural history vouchers, which permits traceability and verification of size measurements. Also, this approach parallels Vermeij’s [*S1-2*] datasets, which were similarly specimen-based. We note how explicit statements about museum vouchers (e.g., museum catalog numbers) in this study differ from analyses that have used fossil marine mammal data to address similar macroecological and evolutionary questions (e.g., [*S3*] and their supplemental materials). This latter study, which was based on a dataset of mammalian body masses (e.g., [*S4*]. MOM v4.1), did not explicitly state data sources (either primary literature or vouchers) in a consistent fashion. This opacity hinders traceability and replication, which is especially important for fossil taxa that occasionally undergo taxonomic changes or revisions to geologic age.

In this study, we collected a broad sample of size measurements on complete specimens within specific ecological guilds of fossil marine mammals; we sought the largest specimen available, either published in the literature or observed in a museum collection, for a given temporal bin, within each geographic region. These maxima are likely not true biological or paleobiological maxima for any one particular taxon, given the remote likelihood of the largest individual from the largest taxon being preserved in the fossil record [see *S5*, for a discussion of this sampling issue]. Similarly, the available voucher record for extant marine mammals does not include the largest individuals known to belong to specific taxa – i.e., we sampled the largest specimen known in museum collections, but in many cases (e.g., for pelagic whaling of mysticetes), much larger specimens lacking vouchers have been reported from field data. Nonetheless, we argue that our survey provides the basis for an adequate outline of the history of body size maxima within these guilds, as confined to specific parameters in time and space.

**2. Data sources**

First, we selected operational taxonomic units (OTUs) from recent, existing phylogenies of these two guilds: for mysticetes, we used Marx and Fordyce [*S6*]; for sirenians, we used Springer et al. [*S7*], and filled in missing fossil dugongids using Velez-Juarbe and Domning [*S8*]; and for desmostylians, we used Beatty [*S9*]. This first step allowed us to generate basic taxonomic bins of candidate genera that were represented by relatively complete cranial material, which we used as a proxy for body size. We also included supplemental data within OTUs (i.e., additional specimens assigned to either species or genera) that have been referred to the taxa by other authors or ourselves. For sirenians, in particular, Sarko et al. [*S10*: Table 4] provided an extensive list of taxa with known skull sizes, which they used for allometric comparisons.

We culled OTUs based on incomplete crania and from geographic regions not covered by either the North Pacific or North Atlantic basins. In the North Pacific, there were no taxonomic occurrences north of the Aleutian Island arc. In the North Atlantic, we included occurrences from countries that currently border this ocean, along with material from the Rhine Basin in continental Europe, but not European or African occurrences bordering the current Mediterranean Sea, which was isolated from the North Atlantic repeatedly during the Neogene.

Additional size data from other specimens, within existing OTUs or from additional ones not included in the matrices were selectively added based on their relative value as maxima within specific temporal or geographic bins. For example, with mysticete cetaceans, we excluded records of “toothed” stem mysticetes (e.g., *Aetiocetus*, *Fucaia*) because of the difficulties in comparing the feeding mode of these taxa with baleen-bearing mysticetes (e.g., crown Mysticeti and some stem mysticetes such as Eomysticetidae, which apparently lack adult dentition). We also added *Cophocetus oregonensis* [*S11*], which, despite being one of the few named mysticetes from the early Miocene in the Northern Hemisphere, is only rarely coded in phylogenetic analyses owing to the incompleteness of the available material. Finally, we added Pleistocene gray whale (*Eschrichtius robustus*) occurrences (e.g., LACM 122322 from the Pacific, and GMNH 27372 from the Atlantic) to provide important maxima within a bin with a sparse intact fossil marine mammal record.

For fossil and modern sirenians, most OTUs are represented by relatively completely crania, which mostly preserve skull length (i.e., condylobasal length), although not always skull width. In some cases, well-represented OTUs, such as *Hydrodamalis gigas*, were represented by complete mandibles instead of crania, and we used these values for poorly sampled intervals (e.g., Pleistocene occurrences of *H. gigas*). This use of mandibular length as a substitute for condylobasal length is justified by a strong correlation between these two measurements, based on an extensive dataset covering living and fossil sirenians [*S12*].

For cetaceans, we used supplementary measurements as surrogates for skull measurements in just one instance: GMNH 27372, a Pleistocene Atlantic gray whale occurrence represented by only a complete mandible [*S13*]. Pyenson et al. [*S14*] demonstrated the close positive allometry between mandible length and total length (TL) in all living mysticetes, and we used this relationship to estimate the TL of GMNH 27372 based on its mandible length. We then looked for a complete, living gray whale specimen with this precise TL among the abundant material available at the Smithsonian Institution and the Natural History Museum of Los Angeles County. We identified LACM 84190, a gray whale specimen with a TL of 9.32 m and a skull width of 90.2 cm.

For desmostylians, there were several instances where both crania and mandibles were available for length measurements, although the greatest common denominator, among osteological elements, are mandibles, which in many cases are relatively complete. Both proxies provided clear evidence of large body forms in specific time bins; whenever both measurements were available for an individual OTU (usually from the same individual specimen), we used the larger of the two measurements. As mentioned in the main text, we presume that allometries similar to those of sirenians constrain desmostylian feeding ecology, given the ecomorphologic similarities in the rostrum and dentition of desmostylians and aquatic sloths (*Thalassocnus* spp.), and to a lesser extent, sirenians.

Lastly, we binned age according to sub-epoch, mostly for comparability with Vermeij’s [*S1-2*] results. Any taxa that ranged through sub-epochs were counted within each sub-epoch; size data were then ordered within each bin. Unless otherwise noted, geologic age determinations followed Marx and Fordyce [*S6*] for mysticetes; and the cited authorities for body size measurements for sirenians and desmostylians.

The Excel spreadsheet included with the ESM documentation for details on specimen data, along with specific comments for reconstructions or surrogate data, where necessary.

**3. Biases**

Uhen and Pyenson [*S15*] provided an extensive examination of the various biases that affect our understanding of the cetacean fossil record. This was the first study on fossil marine mammals that provided a detailed, specimen-based analysis of the factors that overestimate or underestimate our knowledge of the fossil record for these taxa, as part of the broader search for secular patterns of diversity. Uhen and Pyenson [*S15*] also used geologic areal map data for rock outcrops in North America, along with publication and collecting records (where available), to evaluate rock record biases and biases related to study or collecting effort. Using sirenians as a comparative group, Uhen and Pyenson [*S15*] concluded that the fossil record of cetaceans was largely adequate, showing no biases in effort or collecting history, no Pull of the Recent, and mostly lacking major rock outcrop biases (except for the Oligocene, which is poorly sampled). Many of these findings were later corroborated by Marx [*S16*] in a more detailed analysis of the European record for fossil cetaceans and sirenians.

Figure S1a shows geologic map area across sub-epoch bins from the early Eocene through Pliocene of North America, using Uhen and Pyenson’s [*S15*] original data. Middle Eocene rocks, especially from the southeastern US coastal plain, account for the bulk area of Cenozoic rock outcrop. It is worth noting that strong differences between rock area along the eastern and western coasts of the US do exist: for example, abundant early Miocene rocks are known from California and Oregon, whereas similarly aged rocks are far less abundant (by areal map data) along the east coast of the US. Early Oligocene rocks are especially well known from the Pacific Northwest, and they rank among the lowest available rock areas in the dataset, a finding which is generally consistent with the long-standing rarity of fossil marine mammals from this time period.

Using the Paleobiology Database (<http://paleobiodb.org>), we downloaded taxonomic occurrence data for all Cetacea, Sirenia, and Desmostylia in North America (last download 28 February 2016). We binned the data by geologic age (counting range-throughs) in a commensurate way for comparability with rock area (i.e., starting in the early Eocene and to the Pliocene, as Pleistocene rock outcrops were not included in the original dataset [*S15*]), and computed the relative proportion of occurrences for ease of visualization (Fig. S1b). We did not use genera through time (perhaps the most useful taxonomic level for such plots) because we wanted to maximize the size of our datasets, including potential coverage for geologic bins that might not be represented by material worth naming or potentially diagnosable at this taxonomic level. Overall, the patterns of proportional occurrence data for all three groups are remarkably similar, with a notable peak in middle Miocene. A similar pattern has been observed in other richness data (see [*S15*]), indicating that fossil marine mammal occurrences are dense and reliable enough to recover a genuine paleobiological diversity signal.

Comparisons with the available rock area reiterate findings by Uhen and Pyenson [*S15*], which showed that cetacean diversity does not clearly track rock area. Despite the wide exposure of middle Eocene strata, there is no especially large abundance of fossil marine mammal occurrences from this time bin. Fossil sirenians have a more abundant middle Eocene record, but neither have proportional occurrence data on a par with mid-Neogene records. Given that the clear spikes in occurrence data in the middle Miocene for all three groups (a finding paralleled by other diversity data for marine mammals) do not relate to any known bias at this time interval [*S15, S16*], it is likely that they correspond to secular signals in paleodiversity. We argue that their apparent decoupling from body size maxima (Figure 1, Table 1, both in the main text) provides yet more support for the prevalence of physical factors driving evolutionary changes in these ecological groups.

**4. Supplemental text references**

S1 Vermeij GJ. 2011 Shifting sources of productivity in the coastal marine tropics during the Cenozoic era. Proc. Roy. Soc. B 278:2362-2368.

S2 Vermeij GJ. 2012 The evolution of gigantism on temperate seashores. Biol. J. Linn. Soc. 106:776-793.

S3 Evans AR, et al. 2012 The maximum rate of mammal evolution. Proc. Natl. Acad. Sci. USA109:4187-4190.

S4 Smith FA, et al. 2003 Body mass of late Quaternary mammals. Ecology 84:3402

S5 Smith FA, et al. 2010 The evolution of maximum body size of terrestrial mammals. Science 330:1216-1219

S6 Marx FG, Fordyce RE. 2015 Baleen boom and bust: a synthesis of mysticete phylogeny, diversity and disparity. Roy. Soc. Open Sci. 2:140434.

S7 Springer, MS et al. 2015 Interordinal gene capture, the phylogenetic position of Steller’s Sea Cow based on molecular and morphological data, and the macroevolutionary history of Sirenia. Molec. Phylog. Evol. 91:178-193.

S8 Velez-Juarbe J, Domning DP. 2015 Fossil Sirenia of the West Atlantic and Caribbean region. XI. *Callistosiren boriquensis*, gen. et sp. nov. J. Vert. Paleo. 35:e885034.

S9 Beatty BL. 2009 New material of *Cornwallius sookensis* (Mammalia: Desmostylia) from the Yaquina Formation of Oregon. J. Vert. Paleo. 29:894–909.

S10 Sarko DK, Domning DP, Marino L, Reep RL. 2010 Estimating body size of fossil sirenians. Mar. Mamm. Sci. 26:937-959.

S11 Packard EL, Kellogg R. 1934. A new cetothere from the Miocene Astoria Formation of Newport, Oregon. Contributions to Palaeontology, Carnegie Institution of Washington 447:1-62.

S12 Domning DP. 1978 Sirenian evolution in the North Pacific Ocean. Univ. Calif. Pub. Geol. Sci. 118:1-176

S13 Noakes SE, Pyenson ND, McFall G. 2013 Late Pleistocene gray whales (*Eschrichtius robustus*) offshore Georgia, U.S.A., and the antiquity of gray whale migration in the North Atlantic Ocean. Palaeo, Palaeo, 392:502–509.

S14 Pyenson ND, Goldbogen JA, Shadwick RE. 2013 Mandible allometry in extant and fossil Balaenopteridae (Cetacea: Mammalia): the largest vertebrate skeletal element and its role in rorqual lunge feeding. Biol. J. Linn. Soc. 108:586-599.

S15 Uhen MD, Pyenson ND. 2007 Diversity estimates, biases, and historiographic effects: resolving cetacean diversity in the Tertiary. Palaeo. Electronica 10:1-22.

S16 Marx FG. 2009 Marine mammals through time: when less is more in studying palaeodiversity. Proc. Roy. Soc. Lon. B. 276:887-892.

**5. Institutional abbreviations cited in the dataset**

The following institutional abbreviations were used for specimens and their respective citations listed in the Excel spreadsheet included with the ESM documentation:

**AMP**, Ashoro Museum of Paleontology, Ashoro, Hokkaido, Japan; **BMNH**, previous British Museum collections, now at Natural History Museum (**NHM**), London, United Kingdom; **BSP**, Bayerische Staatssammlung für Paläontologie und Geologie München; **CAS**, Department of Invertebrate Zoology and Geology, California Academy of Sciences, San Francisco, USA; **CGM,** Cairo Geological Museum, Cairo, Egypt; **ChM**, The Charleston Museum, Charleston, USA; **CMN**, Canadian Museum of Nature, Ottawa, Canada; **ECOCHM**, Museo de Zoología, El Colegio de la Frontera Sur (ECOSUR), Chetumal, Quintana Roo, Mexico; FGS, **FGS**, former Florida Geological Survey collection, now housed at the Florida Museum of Natural History, see UF; **GMNH (USA),** Georgia Museum of Natural History, Athens, Georgia, U.S.A.; **GMNH (Japan)**, Gunma Museum of Natural History, Tomioka, Japan; **HMN**, Hiwa Museum of Natural History, Hiwa, Japan; **GSJ**, Geological Survey of Japan, Tsukuba, Japan; **IGM**, Institute de Geologiade la Universidad Nacional Autonomo de Mexico, Mexico City, Mexico; **IRSNB**, Institut Royal des Sciences Naturelles de Belgique, Brussels, Belgium; **KMNH**, Kitakyushu Museum of Natural and Human History, Kitakyushu, Kyushu, Japan; **LACM**, Natural History Museum of Los Angeles County, Los Angeles, USA; **MFM**, Mizunami Fossil Museum, Gifu, Japan; **MSM,** Museum Sønderjylland, Department of Natural History and Paleontology, Gram, Denmark; **MOTA,** Museum of the Aleutians, Unalaska, Alaska, USA; **MVZ**, Museum of Vertebrate Zoology, University of California, Berkeley, California, U. S. A.;, **NHG**, Natuurhistorische collectie van het Zeeuws Genootschap der Wetenschappen, Middelburg, the Netherlands; **NMB**, Natuurmuseum Brabant, Tilburg, The Netherlands; **NMST**, National Museum of Nature and Science, Tokyo, Japan; **SBNHM**, Santa Barbara Museum of Natural History, Santa Barbara, California, USA; **SDSNH**, San Diego Natural History Museum, San Diego, USA; **SFM**, Shinshushinmachi Fossil Museum, Shinshushinmachi, Japan; **SMNH**, Saitama Museum of Natural History, Saitama, Japan; **STMBV**, Stadtmuseum Bad Vöslau, Lower Austria, Austria; **UCMP**, University of California Museum of Paleontology, Berkeley, California, USA; **UF**, Florida Museum of Natural History, Gainesville, Florida, USA; **UHR**, University of Hokkaido Registration, Sapporo, Japan; **UO**, Museum of Natural and Cultural History, University of Oregon, Eugene, Oregon, USA; **USNM**, National Museum of Natural History, Smithsonian Institution, Washington DC, USA; **Yamagata**, Yamagata Prefectural Museum, Japan.

**6. References cited in the dataset**

**Cetacea**

S17 Bisconti M. 2005 Skull morphology and phylogenetic relationships of a new diminutive balaenid from the Lower Pliocene of Belgium. Palaeontology 48:793-816.

S18 Bisconti M, Lambert O, Bosselaers M. 2013 Taxonomic revision of *Isocetus depauwi* (Mammalia, Cetacea, Mysticeti) and the phylogenetic relationships of archaic cetothere mysticetes. Palaeontology 56:95-127.

S19 Bosselaers M, Post K. 2010 A new fossil rorqual (Mammalia, Cetacea, Balaenopteridae) from the Early Pliocene of the North Sea, with a review of the rorqual species described by Owen and Van Beneden. Geodiversitas 32:331-363.

S20 Case EC. 1904 Mammalia (ed. WB Clark), pp. 3-57. Baltimore: The John Hopkins Press.

S21 Cope, ED. 1895 Fourth contribution to the marine fauna of the Miocene period of the United States. Proc. Am. Philos. Soc. 34:135-155.

S22 Cope, ED. 1896 Sixth contribution to the knowledge of the Miocene fauna of North Carolina. Proc. Am. Philos. Soc. 35:139–146.

S23 Deméré TA, Berta A, McGowen MR. 2005 The taxonomic and evolutionary history of fossil and modern balaenopteroid mysticetes. J. Mamm. Evol. 12 (1-2):99-143.

S24 El Adli JJ, Deméré TA, Boessenecker RW. 2014 *Herpetocetus morrowi* (Cetacea: Mysticeti), a new species of diminutive baleen whale from the Upper Pliocene (Piacenzian) of California, USA, with observations on the evolution and relationships of the Cetotheriidae. Zool. J. Linn. Soc. Lond. 170:400-466.

S25 Kellogg R. 1922 Description of the skull of *Megaptera miocaena*, a fossil humpback whale from the Miocene diatomaceous earth of Lompoc, California. Proc. US Natl. Mus. 61:1-18.

S26 Kellogg R. 1924 Description of a new genus and species of whalebone whale from the Calvert Cliffs, Maryland. Proc. US Natl. Mus. 63:1-14.

S27 Kellogg R. 1929 A new cetothere from southern California. Univ. Calif. Pub. Geol. Sci. 18:449-457.

S28 Kellogg R. 1931 Pelagic mammals of the Temblor Formation of the Kern River region, California. Proc. Calif. Acad. Sci. 19:217-397.

S29 Kellogg R. 1965 Fossil marine mammals from the Miocene Calvert Formation of Maryland and Virginia, part 1: a new whalebone whale from the Miocene Calvert Formation. Bull. US Natl. Mus. 247:1-45.

S30 Kellogg R. 1968 Fossil marine mammals from the Miocene Calvert Formation of Maryland and Virginia, part 5: Miocene Calvert mysticetes described by Cope. Bull. US Natl. Mus. 247:103-132.

S31 Kellogg, R. 1968 Fossil marine mammals from the Miocene Calvert Formation of Maryland and Virginia, part 6: a hitherto unrecognized Calvert cetothere. Bull. US Natl. Mus. 247:133-161.

S32 Kellogg, R. 1968 Fossil marine mammals from the Miocene Calvert Formation of Maryland and Virginia, part 7: a sharp-nosed cetothere from the Miocene Calvert. Bull. US Natl. Mus. 247:163–173.

S33 Kellogg R. 1968 Fossil marine mammals from the Miocene Calvert Formation of Maryland and Virginia, part 8: supplement to the description of *Parietobalaena palmeri*. Bull. US Natl. Mus. 247:175-197.

S34 Kellogg R. 1969 Cetothere skeletons from the Miocene Choptank Formation of Maryland and Virginia. Bull. US Natl. Mus. 294:1-40.

S35 Kimura T, Ozawa T. 2002 A new cetothere (Cetacea: Mysticeti) from the early Miocene of Japan. J. Vert. Paleo. 22:684-702.

S36 Kimura T. 2009 Review of the fossil balaenids from Japan with a re-description of *Eubalaena shinshuensis* (Mammalia, Cetacea, Mysticeti). Quad. Mus. Stor. Nat. Livorno. 22:3-21.

S37 Kimura T, Hasegawa Y. 2010 A new baleen whale (Mysticeti: Cetotheriidae) from the earliest late Miocene of Japan and a reconsideration of the phylogeny of cetotheres. J Vert. Paleo. 30:577-591.

S38 Noakes SE, Pyenson ND, McFall G. 2013 Late Pleistocene gray whales (*Eschrichtius robustus*) offshore Georgia, U.S.A., and the antiquity of gray whale migration in the North Atlantic Ocean. Palaeo. Palaeo. Palaeo. 392:502–509.

S39 Okazaki Y. 2012 A new mysticete from the upper Oligocene Ashiya Group, Kyushu, Japan and its significance to mysticete evolution. Bull. Kitakyushu Mus. Nat. Hist. Ser. A. 10:129-152.

S40 Otsuka H, Ota Y. 2008 Cetotheres from the early Middle Miocene Bihoku Group in Shobara District, Hiroshima Prefecture, West Japan. Misc. Rep. Hiwa Mus. Nat. Hist. 49:1-66.

S41 Plisnier-Ladame F, Quinet GE. 1969 *Balaena belgica* Abel 1938, cetace du merxemien d‟Anvers. Bull. Inst. Roy. Sci. Nat. Belgique Biol. 45:1-6.

S42 Sanders AE, Barnes LG. 2002a Paleontology of the Late Oligocene Ashley and Chandler Bridge Formations of South Carolina, 2: *Micromysticetus rothauseni*, a primitive cetotheriid mysticete (Mammalia: Cetacea). Smithsonian Contrib. Paleo. 93:271-293.

S43 Sanders AE, Barnes LG. 2002b. Paleontology of the Late Oligocene Ashley and Chandler Bridge Formations of South Carolina, 3: Eomysticetidae, a new family of primitive mysticetes (Mammalia: Cetacea). Smithsonian Contrib. Paleo. 93:313-356

S44 Steeman ME. 2007 Cladistic analysis and a revised classification of fossil and recent mysticetes. Zool. J. Linn. Soc. Lon. 150:875-894.

S45 Steeman ME. 2009 A new baleen whale from the Late Miocene of Denmark and early mysticete hearing. Palaeontology 52:1169-1190.

S46 Westgate JW, Whitmore FC, Jr. 2002 *Balaena ricei*, a new species of bowhead whale from the Yorktown Formation (Pliocene) of Hampton, Virginia. Smithsonian Contrib. Paleo. 93:295–312.

S47 Whitmore FC, Jr, Barnes LG. 2008 The Herpetocetinae, a new subfamily of extinct baleen whales (Mammalia, Cetacea, Cetotheriidae). Va. Mus. Nat. Hist. Special Pub. 14:141-180.

S48 Whitmore FC, Jr., Kaltenbach JA. 2008 Neogene Cetacea of the Lee Creek Phosphate Mine, North Carolina. Va. Mus. Nat. Hist. Special Pub. 14:181-269.

S49 Yoshida K, Kimura T, Hasegawa, Y. 2003 New cetothere (Cetacea: Mysticeti) from the Miocene Chichibumachi Group, Japan. Bull. Saitama Mus. Natl. Hist. 20-21:1-10.

S50 Zeigler CV, Chan GL, Barnes LG. 1997 A new Late Miocene balaenopterid whale (Cetacea: Mysticeti), *Parabalaenoptera baulinensis*, (new genus and species) from the Santa Cruz Mudstone, Point Reyes Peninsula, California. Proc. Calif. Acad. Sci. 50:115- 138.

**Sirenia and Desmostylia**

S51 Barnes LG. 2013. A new genus and species of late Miocene paleoparadoxiid (Mammalia, Desmostylia) from California. Los Angeles County Mus. Contrib. Sci. 521:51–114.

S52 Beatty BL. 2009. New material of *Cornwallius sookensis* (Mammalia: Desmostylia) from the Yaquina Formation of Oregon. J. Vert. Paleo. 29:894–909.

S53 Beatty BL, Geisler J. 2010 A stratigraphically precise record of *Protosiren* (Protosirenidae, Sirenia) from North America. Neues Jahrb. Geol. Paläontol. Abh. 258:185–194.

S54 Domning DP. 1978 Sirenian evolution in the North Pacific Ocean. Univ. Calif. Pub. Geol. Sci. 118:1-176.

S55 Domning DP. 1988 Fossil Sirenia of the west Atlantic and Caribbean region. I. *Metaxytherium floridanum* Hay, 1922. J. Vert. Paleo. 8:395-426.

S56 Domning DP. 1989 Fossil Sirenia of the west Atlantic and Caribbean region. II. *Dioplotherium manigaulti* Cope, 1883. J. Vert. Paleo 9:415-428.

S57 Domning DP. 1989 Fossil Sirenia of the West Atlantic and Caribbean region. III. *Xenosiren yucateca*, gen. et sp. nov. J. Vert. Paleo. 9:429-437.

S58 Domning DP. 1990 Fossil Sirenia of the West Atlantic and Caribbean Region. IV. *Corystosiren varguezi*, gen. et sp. nov. J. Vert. Paleo. 10:361-371.

S59 Domning DP. 2005. Fossil Sirenia of the West Atlantic and Caribbean region. VII. Pleistocene *Trichechus manatus* Linnaeus, 1758. J. Vert. Paleo. 25:685-701.

S60 Domning DP, Aguilera OA. 2008 Fossil Sirenia of the West Atlantic and Caribbean region. VIII. *Nanosiren garciae*, gen. et sp. nov. and *Nanosiren sanchezi*, sp. nov. J. Vert. Paleo. 28:479-500.

S61 Domning DP, Gingerich PD. 1994 *Protosiren smithae*, new species (Mammalia, Sirenia), from the late middle Eocene of Wadi Hitan, Egypt. Contrib. Mus. Paleo. Univ. Mich. 29:69-87.

S62 Domning DP, Pervesler P. 2012 The sirenian *Metaxytherium* (Mammalia: Dugongidae) in the Badenian (Middle Miocene) of Central Europe. Austrian J. Earth Sci. 105:125-160.

S63 Domning DP, Ray CE, McKenna MC. 1986. Two new Oligocene desmostylians and a discussion of Tethytherian systematics. Smithsonian Contrib. Paleo. 59:1-56.

S64 Kellogg R. 1966 New species of extinct Miocene Sirenia. Bull. US Natl. Mus. 247: 65-98*.*

S65 Kilmer FH. 1965 A Miocene dugongid from Baja California, Mexico. Bull. Southern Calif. Acad. Sci. 65:57–74.

S66 Reinhart RH. 1959 A review of the Sirenia and Desmostylia. Univ. Calif. Pub. Geol. Sci. 16:1–146.

S67 Reinhart RH. 1976 Fossil sirenians and desmostylids from Florida and elsewhere. Bull. Florida State Mus. Biol. Sci. 20:187-300

S68 Sagne C. 2001 *Halitherium taulannense,* nouveau sirénien (Sirenia, Mammalia) de l’Éocène supérieur provenant du domaine Nord-Téthysien (Alpes-de-Haute-Provence, France). C. R. Acad. Sci. Paris, Sci. Terre planètes / Earth and Planetary Sciences 333:471–476

S69 Sarko DK, Domning DP, Marino L, Reep RL. 2010 Estimating body size of fossil sirenians. Mar. Mamm. Sci. 26:937-959.

S70 Savage RJG, Domning DP, Thewissen JGM. 1994 Fossil Sirenia of the West Atlantic and Caribbean region. V. The most primitive known sirenian, *Prorastomus sirenoides* Owen, 1855. J. Vert. Paleo. 14:427-449.

S71 Uno H, Kimura M. 2004 Reinterpretation of some cranial structures of *Desmostylus hesperus* (Mammalia: Desmostylia): a new specimen from the Middle Miocene Tachikaraushinai Formation, Hokkaido, Japan. Paleontol. Res. 8:1–10.

S72 Velez-Juarbe J, Domning DP, Pyenson ND. 2012 Iterative evolution of sympatric seacow (Dugongidae, Sirenia) assemblages during the past∼ 26 million years. PLoS One 7:e31294.

S73 Velez-Juarbe J, Domning DP. 2014 Fossil Sirenia of the West Atlantic and Caribbean region: X. *Priscosiren atlantica*, gen. et sp. nov., J. Vert. Paleo. 34:951-964.

S74 Velez-Juarbe J, Domning DP. 2015 Fossil Sirenia of the West Atlantic and Caribbean region. XI. *Callistosiren boriquensis*, gen. et sp. nov. J. Vert. Paleo. 35:e885034.

S75 Whitmore FC, Jr, Gard LM. 1977 Steller's sea cow (*Hydrodamalis gigas*) of late Pleistocene age from Amchitka, Aleutian Islands, Alaska. No. 1036 US Govt. Print. Off.

**7. Figure S1.** (a) Rock outcrop area, in square kilometers, for North American marine rock units, using unpublished data collected for analyses published in Uhen & Pyenson [*S15*], binned by available sub-epochs (note lack of post-Pliocene data). (b) Taxonomic occurrences of cetaceans, sirenians, and desmostylians, by percetange of total occurrence records, binned to the same time span as (a).

**
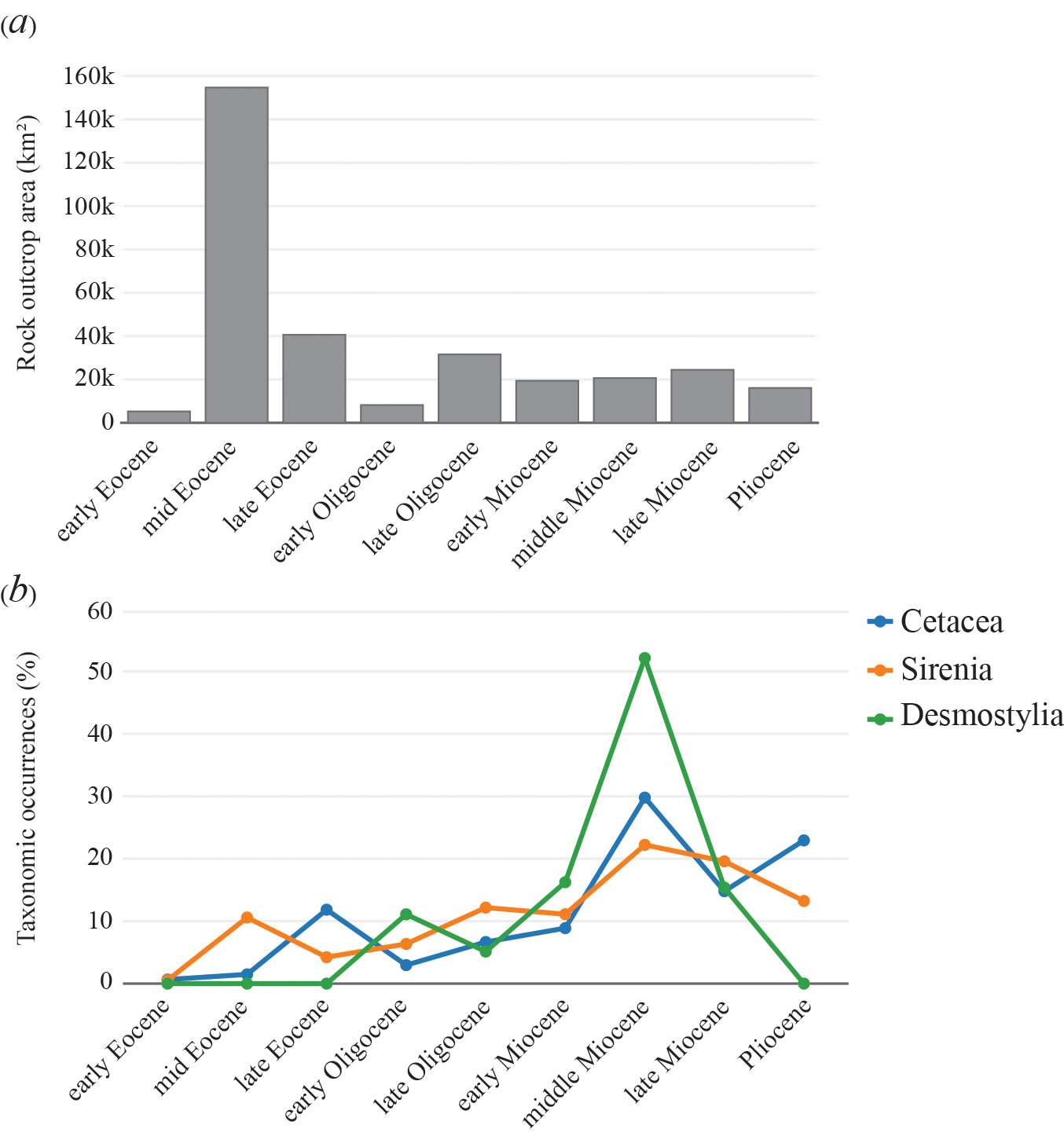
**
